# Elastosonographic features of the metacarpophalangeal joint capsule in horses

**DOI:** 10.1186/s12917-021-02897-8

**Published:** 2021-05-29

**Authors:** Paola Straticò, Giulia Guerri, Adriana Palozzo, Paola Di Francesco, Massimo Vignoli, Vincenzo Varasano, Lucio Petrizzi

**Affiliations:** Faculty of Veterinary Medicine, University of Teramo, Loc. Piano D’Accio, 64100 Teramo, Italy

**Keywords:** Elasticity, Elastosonography, Metacarpophalangeal joint, Joint capsule, Horse, Osteoarthritis

## Abstract

**Background:**

Capsulitis leads to the release of inflammatory mediators in the joint, causing capsular fibrosis and osteoarthritis (OA). Strain elastosonography (SE) measures the elasticity of tissue by evaluating its strain in operator-dependent deformation. The aims of the study were to assess the feasibility, repeatability, and reproducibility of SE for imaging the distal attachment of the joint capsule (DJC) of metacarpophalangeal joints in sound horses (Group S) and in horses with metacarpophalangeal OA (Group P) and to evaluate differences in the elastosonographic patterns of these horses. After a whole lameness examination, fore fetlock DJCs were assigned to Group S and Group P and were thereafter examined by two operators using SE. Qualitative (i.e., colour grading score) and semi-quantitative (i.e., elasticity index (EI) and strain ratio (SR)) methods were used to evaluate the elastograms. The inter-rater reliability (IRR), intraclass correlation coefficient (intra-CC) and interclass correlation coefficient (inter-CC) were used to compare colour grading scores and the repeatability and reproducibility of EI and SR outcomes. The same parameters were compared between groups. *P* < 0.05 indicated a significant finding.

**Results:**

Forty-one horses were included: 11 were in Group S and 30 were in Group P (16 with bilateral OA, 8 with left OA and 6 with right OA). IRR outcomes ranged from good to excellent. For transverse and longitudinal ultrasound scans, the colour grading score of Group S was significantly higher than the metacarpophalangeal DJCs of Group P. Both Inter-CC and intra-CC were higher in Group S than in Group P, with values always > 0.8. Significative differences in EI and SR were detected between groups and between Group S and the affected limb of Group P; values were lower in Group S than in Group P.

**Conclusions:**

SE can be a useful technique for evaluating DJCs, with good repeatability and reproducibility. DJCs appear softer in sound horses.

**Supplementary Information:**

The online version contains supplementary material available at 10.1186/s12917-021-02897-8.

## Background

Elastosonography is an ultrasound-based technique that measures tissue stiffness and is used to evaluate the mechanical properties of tissues, providing information complementary to B-mode imaging [1, 2]. Strain elastosonography (SE) measures the relative strain of one area compared to another as external stress is applied by the ultrasound probe. This strain is displayed as an elastogram superimposed on a B-mode image, which allows for a qualitative and semi-quantitative analysis of the target tissue, the calculation of its Elasticity Index (EI) and the Strain Ratio (SR) between the tissue and a reference region, which is likely to experience the same degree of stress [3].

Due to the release of inflammatory mediators into the synovial cavity, synovitis and capsulitis have been recognized as clinical features of osteoarthritis (OA) and as drivers of this process [4]. In addition to producing pain and discomfort in horses, these features indeed contribute to the degenerative process in articular cartilage by the release of enzymes, inflammatory mediators and cytokines. Moreover, chronic capsulitis and synovitis lead to changes in tissue composition and increased fibrosis of the joint capsule and soft tissue surrounding the joint [5].

SE was used successfully to evaluate the metacarpal tendons in clinically normal horses and in horses with naturally occurring tendinopathies and desmopathies [6–9]. To the author’s knowledge, this is the first study to assess the feasibility of SE for evaluating the stiffness of the distal attachment of the metacarpophalangeal joint capsules (DJCs) in sound horses and in horses with OA and to assess whether SE can be used to detect a reduction in elasticity of the DJC. The repeatability and reproducibility of the technique were assessed, and the appearance of the region using strain elastosonography in both groups was described.

## Materials and methods

All study procedures were approved by the local Ethical Committee (Prot. 11/2019). Horses presented at the Veterinary Teaching Hospital (VTH) of the University of Teramo were prospectively recruited. Horse owners were aware of the procedure that was going to be undertaken and signed an informed consent form. Adult horses of both sexes and different breeds underwent a whole orthopaedic evaluation by a certified equine surgeon (LP). Horses free from lameness, undergoing a radiographic and ultrasonographic examination of fore fetlocks, were included in group S. Horses that had a positive low ring block and/or intra-articular anaesthesia of the metacarpophalangeal joint after a lameness examination were included in group P [10]. After the localization of the lameness, radiographic and ultrasonographic examinations of both metacarpophalangeal joints were undertaken (group P).

For diagnostic imaging, all horses were sedated with xylazine 0.5 mg/kg (Nerfasin, ATI) intravenously. Fore fetlock joints were examined to assess the presence of abnormal findings of osseous or soft tissue structures [11]. To facilitate comparisons between sound horses and horses affected by different degrees of metacarpophalangeal OA, radiographic and ultrasonographic joint scores were assigned to each joint. The radiographic score was assigned based on the evaluation of osteophytosis development in a four-degree system for judgement (0–3), corresponding to a progressive worsening stage of OA [12]. Osteochondral irregularities, an increased and heterogeneous plica, and an increased thickness of the joint capsule were evaluated to assign the ultrasonographic score using a scale ranging from 0 to 14 points, modified from Yamada and its colleagues [13] [Supplementary material].

Radiographic examinations were performed with a M.T. Medical Technology CS01MS equipment in latero-medial and dorso-palmar views; for the ultrasonographic exams, a high frequency linear probe (8.5–10 MHz) connected to an ultrasound system (Logiq S8XD Clear, GE) was used.

Elastosonography of the DJC at the dorsal aspect of the proximal phalanx (P1) was performed by two experienced operators in transverse and longitudinal ultrasound scans with the limb in a weight-bearing position. Each operator executed rhythmic and regular low-frequency compression and relaxation cycles with the probe above the area of interest. To obtain an appropriate image, the pressure exerted was moderate (i.e., level of pressure needed to maintain contact with skin); very high or low pressure was avoided, as the elastic properties of tissue became nonlinear [14]. To ensure the application of the correct pressure, the force applied to the area was adjusted according to the strain-bar indicator on the lateral part of the elastogram. Moreover, to minimize inter-operator variations, each SE scan was repeated several times until at least three compression-decompression cycles of optimal strain were obtained at the region of interest. Elastosonographic images were independently and randomly analysed by two observers who were blinded to the group to which the horses were assigned. Qualitative and semi-quantitative methods were used to evaluate the elastograms [14]. The region of interest (ROI) (2 mm circumference) was placed over the DJC for the qualitative assessment and over the dorsal digital extensor tendon (DDET) as a reference region for the semi-quantitative assessment. For the qualitative analysis, a colour grading score assessment of DJC was performed, assigning a score ranging from 1 to 5 to DJC (1 = blue; 2 = green; 3 = yellow; 4 = orange; 5 = red) [15], where blue encodes hard DJC and red encodes soft DJC. For the semi-quantitative assessment, the elastosonographic software calculated the EI of the DJC and the SR between the DJC and the DDET.

To assess intra-operator agreement, every measurement was taken three times by each operator, and the data were analysed for normality using the Shapiro-Wilk test. For qualitative analysis, inter-operator agreement on the same image was calculated using the inter-rater reliability (IRR) test [16]. For the semi-quantitative analysis, the Mann-Whitney U test and the Friedman test were used to compare data collected by the two operators or by the same operator, respectively. Wilcoxon test compared left to right limbs. To assess the inter- and intra-observer agreement, the interclass correlation coefficient (inter-CC) and intraclass correlation coefficient (intra-CC) were also calculated for EI and SR in both groups. Intra-CC estimated the overall correlation between the three measurements taken by a single operator, while the Inter-CC estimated the overall correlation of the measurements between two observers; they were classified as poor (0.00-0.20 CC), fair (0.20–0.40 CC), good (0.40–0.75 CC) and excellent (> 0.75 CC) [16]. Colour grading score, EI and SR were compared among Group S and Group P with the Mann-Whitney U test.

Data were collected on digital worksheets (Excel, Microsoft) and analysed with statistical software (SPSS V.25.0, IBM). Statistical significance was determined by *P* values < 0.05.

## Results

Forty-one horses were included in the study, with a median age of 10 years (2–20 years); 25 were males (7 stallions, 18 geldings), and 16 were females.

Eleven horses were assigned to Group S. They were represented by 7 males (2 stallions, 5 geldings) and 4 females, with a median age of 8.5 years (5–20 years). They were 4 eventers, 4 western riding horses, and 3 hacking horses.

Thirty horses were allocated to Group P. Eighteen were males (5 stallions, 13 geldings), and 12 were females with a median age of 10 years (2–20 years). Thirteen were western riding horses, 8 eventers, 3 hacking horses, 3 endurance horses, 2 trotters and 1 racehorse. Sixteen horses showed bilateral OA involvement, 8 showed left metacarpophalangeal OA and 6 showed right metacarpophalangeal OA. The mean radiographic scores were 1.7 ± 0.73 on the left forelimb and 1.33 ± 0.57 on the right forelimb. Only moderate sclerosis and osteophytosis were observed in all the horses of group P, except three horses, where severe OA (i.e., score 3) was present. Concerning the ultrasonographic examination outcomes, the mean total scores were 3.96 ± 1.95 on the left forelimb and 3.03 ± 1.55 on the right forelimb. Osteochondral irregularities were reported in twenty-one horses, with a mean score of 0.85 ± 0.85. Increased and heterogeneous plica was observed in twenty-six horses, with mean scores of 0.57 ± 0.66 and 0.71 ± 0.66. The joint capsule was thickened in twenty-three horses and hypoechoic in twenty-nine horses, with mean scores of 0.66 ± 0.61 and 0.87 ± 0.50, respectively. Twenty-seven horses presented moderately irregular joint capsule insertion, with a mean score of 0.87 ± 0.50.

### Qualitative analysis

The mean colour grading scores of Group S and of the affected and unaffected limbs of Group P are summarized in Table 1.

**Table 1:** Colour grading score of the Group S and of the affected and unaffected limbs of the Group P

	Transverse scan	Longitudinal scan
Group S	4.79 ^a^	4.95 ^a^
Group P	3.6 ^bc^	3.8 ^bc^
Affected limb in the Group P	3.68 ^b^	3.78 ^b^
Not affected limb in the Group P	3.75 ^c^	3.84 ^c^

Different letters in the same column indicate significantly different results (a, b, c: *P* < 0.05)

**Fig. 1:**
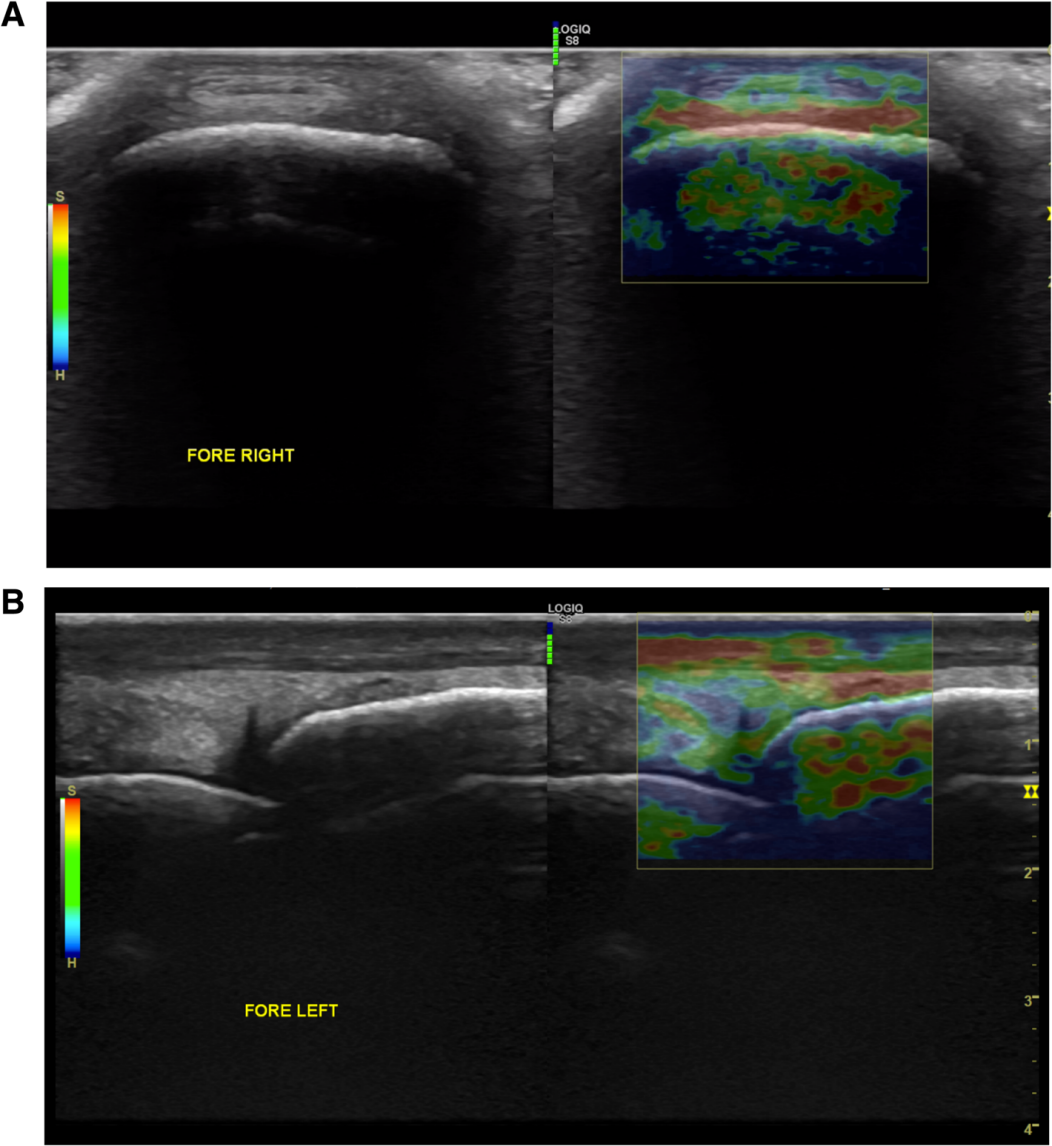
Transverse (**a**) and longitudinal (**b**) ultrasound scans of the distal attachment of the fetlock joint capsule (DJC) in one horse of Group S. The elastogram superimposed on the B-mode images shows a reddish colouration of the DJC on the transverse image and a green colouration on the longitudinal scan (red dotted line) (P1: proximal phalanx; McIII: third metacarpal bone; DDET: dorsal digital extensor tendon; DJC: distal attachment of the joint capsule)

In Group S, the IRR was excellent for both the transverse and longitudinal views (0.8 and 0.85, respectively), with mean colour grading scores of the DJC of 4.79 and 4.95, respectively. The cross-sectional and longitudinal ultrasound images of the distal attachment of the fetlock joint capsule (DJC) in one horse of Group S are displayed in Fig. 1 a, b.

**Fig. 2:**
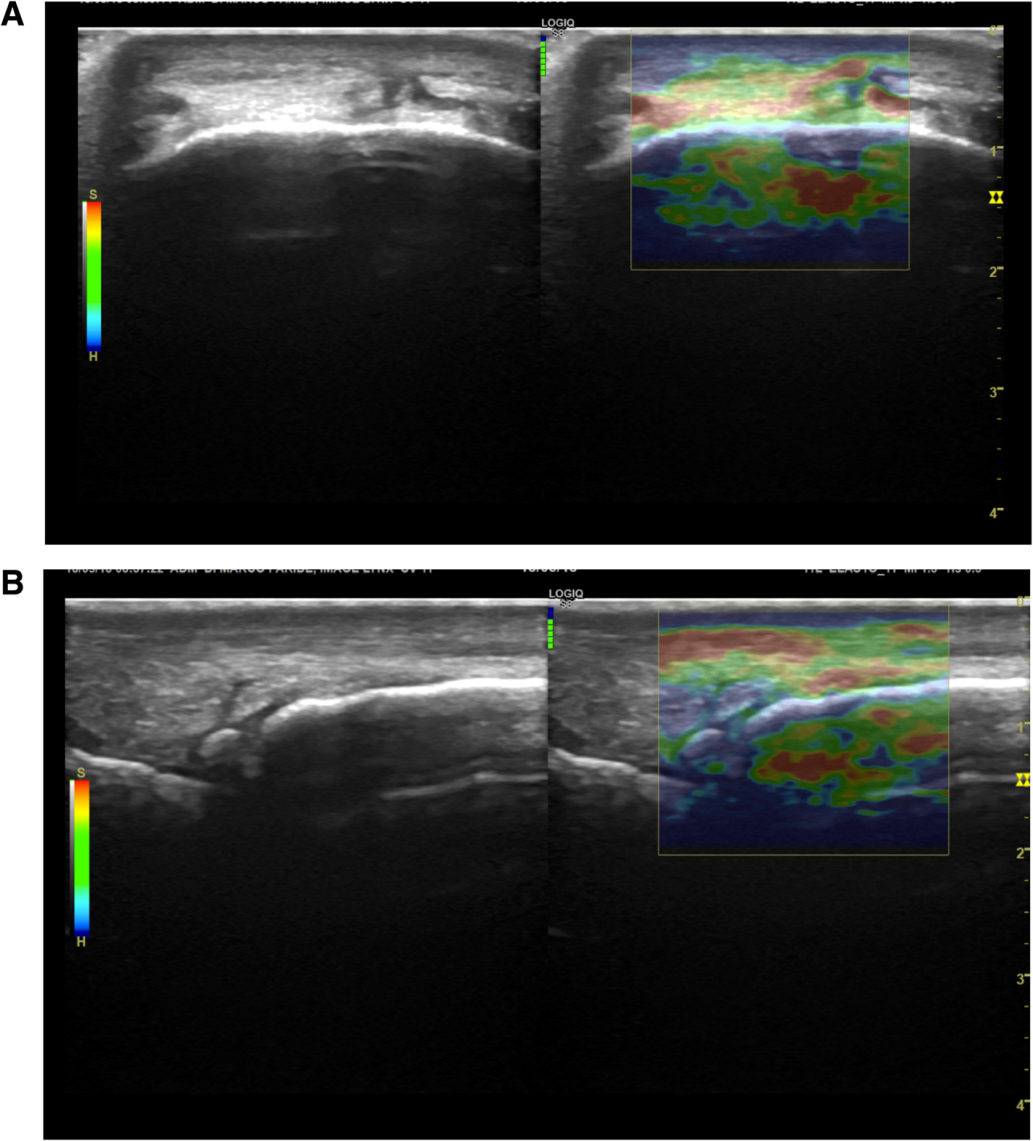
Transverse (**a**) and longitudinal (**b**) ultrasound scans of the distal attachment of the fetlock joint capsule (DJC) in one horse of Group P. The elastogram superimposed on the B-mode images shows green colouration of the DJC on the transverse image and green-blue colouration on the longitudinal image (red dotted line) (P1: proximal phalanx; McIII: third metacarpal bone; DDET: dorsal digital extensor tendon; DJC: distal attachment of the joint capsule). On the longitudinal scan, a dorsal osteochondral fragment of the proximal phalanx (P1) is visible (yellow dot)

In Group P, the IRR was classified as good in transverse and longitudinal scans (0.56 and 0.59, respectively), with mean colour grading scores of the DJC of 3.6 and 3.8, respectively in transverse and longitudinal scans. In the same group (P), the affected limb had colour grading scores of 3.68 and 3.78 respectively in transverse and longitudinal scans, while the unaffected limb scores were 3.75 and 3.84. The cross-sectional and longitudinal ultrasound images of the distal attachment of the fetlock joint capsule (DJC) in one horse of Group P are displayed in Fig. 2 a, b.

The colour grading score of Group S was significantly higher than that of the metacarpophalangeal DJCs of Group P in the affected and unaffected limbs for both scans (*P* < 0.05).

A significant difference was also found when the affected and unaffected limbs of Group P were compared (*P* < 0.05), with lower values in the affected limb in both scans.

### Semi-quantitative analysis

The mean EI and SR values for Group S and Group P in the affected and unaffected limbs of Group P are summarized in Table 2.

**Table 2:** Mean EI and SR values for Group S and Group P for the affected and unaffected limbs of Group P. Values are expressed as the mean ± sd and minimum and maximum values (*EI* elasticity index, *SR* strain ratio, *sd* standard deviation of the mean)

	EI		SR	
	**Transverse**	**Longitudinal**	**Transverse**	**Longitudinal**
**Group S**	0.57 ± 0.19^a^ (0.3–1.3)	0.51 ± 0.14^a^ (0.3-2)	0.32 ± 0.19^a^ (0.1–0.8)	0.28 ± 0.2^a^ (0.2–1.2)
**Group P**	1.09 ± 1^b^ (0.3–5.5)	0.89 ± 0.7^b^ (0.3–4.1)	0.78 ± 1.48^b^ (0.1–13.6)	0.72 ± 0.85^b^ (0.1–5.2)
**Affected limb of P**	1.05 ± 0.95^b^ (0.3–4.9)	0.83 ± 0.67^b^ (0.3–4.1)	0.7 ± 1.05^b^ (0.1–7.1)	0.68 ± 0.72^b^ (0.1-7)
**Unaffected limb of P**	0.85 ± 0.91^ab^ (0.3–5.5)	0.77 ± 0.62^c^ (0.1-7)	0.71 ± 1.87^ab^ (0.1–13.6)	0.57 ± 0.67^c^ (0.1–4.1)

Different letters in the same column indicate significantly different results (a, b, c: *P* < 0.05)

In Group S, the mean EI was 0.57 ± 0.19 in the transverse scans and 0.51 ± 0.14 in the longitudinal scans. The mean SRs were 0.32 ± 0.19 and 0.28 ± 0.2 respectively in the transverse and longitudinal images. In Group P, the mean EI in the transverse scans was 1.09 ± 1, and in the longitudinal scans, it was 0.89 ± 0.7; the mean SRs were 0.78 ± 1.48 and 0.72 ± 0.85 respectively in the transverse and longitudinal images.

In Group P, the affected limbs had a mean EI value of 1.05 ± 0.95 and 0.83 ± 0.67 in transverse and longitudinal scans, while the unaffected limbs had a mean EI value of 0.85 ± 0.91 and 0.77 ± 0.62.

In Group P, the affected limbs had mean SR values of 0.7 ± 1.05 and 0.68 ± 0.72 respectively in transverse and longitudinal images, whereas the unaffected limbs had mean SR values of 0.71 ± 1.87 and 0.57 ± 0.67, respectively.

**Fig. 3:**
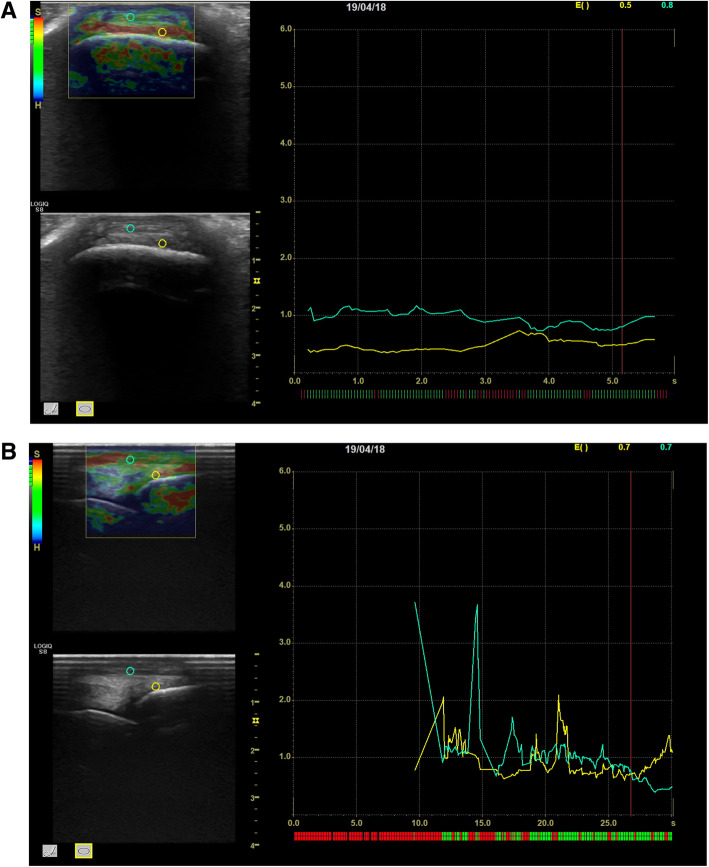
Transverse (**a**) and longitudinal (**b**) elastosonographic images of the distal attachment of the fetlock joint capsule (DJC) showing the positioning of the ROIs over the DJC (yellow circle) and the dorsal digital extensor tendon (DDET) (green circle) in one horse of Group S. The top left box shows the elastogram of the transverse (**a)** and longitudinal (**b**) scans of the DJC with the superimposed ROI above the DJC (yellow) and DDET (green) in Group S. The bottom left box shows the conventional B-mode image of a transverse and a longitudinal scan of the DJC with the superimposed ROI above the DJC (yellow) and DDET (green). The box at the right side of the image shows the graph produced by the software: the x-axis is time-dependent with green segments indicating the moments of proper pressure and red segments the incorrect ones. The y-axis shows the EI values for the DJC (yellow) and DDET (green). The values of EI are expressed at the top right side of the image. SR, not shown, is then calculated by the software (ROI: region of interest; DJC: distal attachment of the joint capsule; DDET: dorsal digital extensor tendon; EI: Elasticity Index; SR: Strain Ratio)

**Fig. 4:**
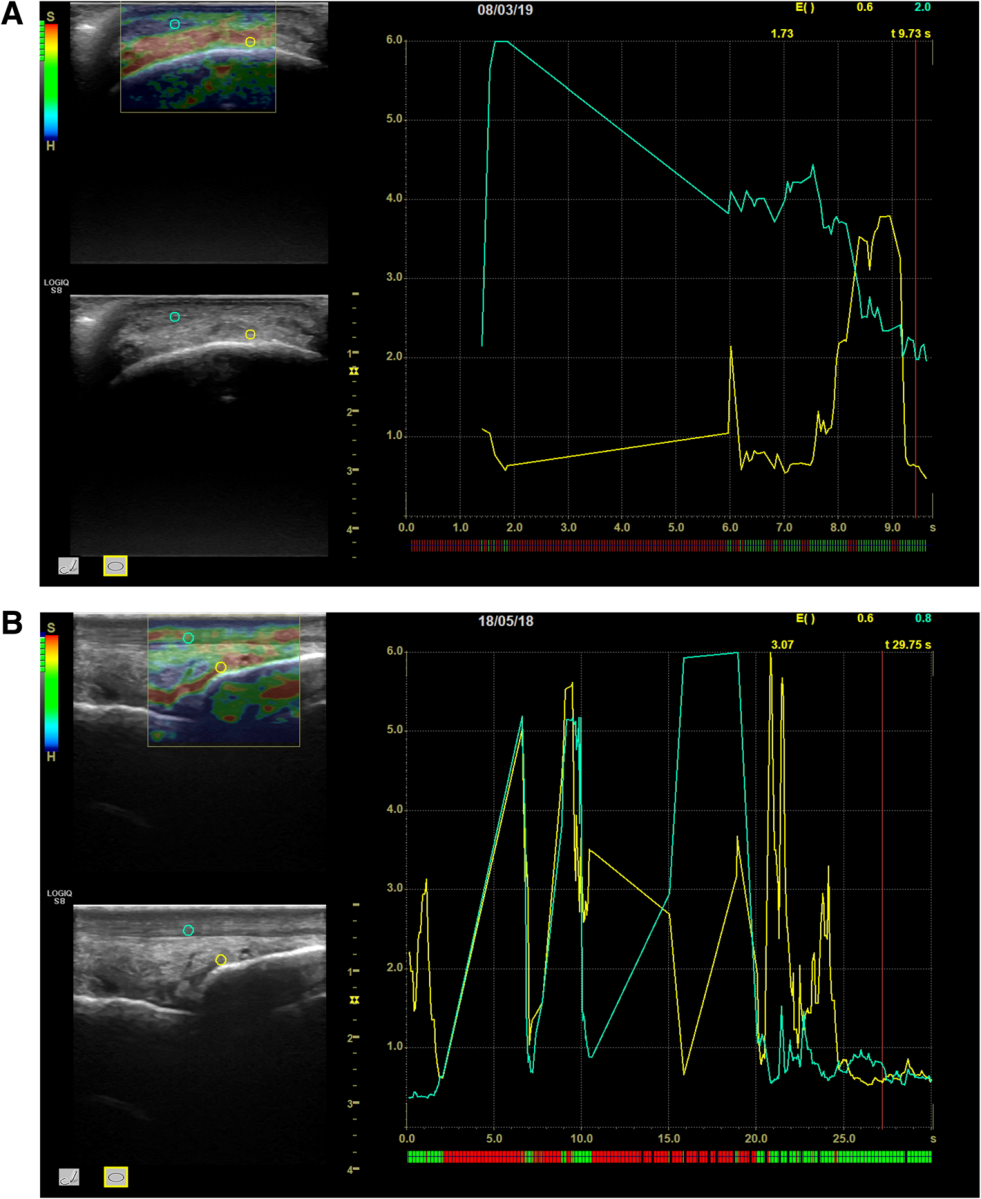
Transverse (**a**) and longitudinal (**b**) elastosonographic images of the distal attachment of the fetlock joint capsule (DJC) showing the positioning of the ROIs over the DJC (yellow circle) and the dorsal digital extensor tendon (DDET) (green circle) in one horse of Group P. The top left box shows the elastogram of the transverse (**a**) and longitudinal (**b**) scans of the DJC with the superimposed ROI above the DJC (yellow) and DDET (green) in group P. The bottom left box shows the conventional B-mode image of a transverse and a longitudinal scan of the DJC with the superimposed ROI above the DJC (yellow) and DDET (green). The box at the right side of the image shows the graph produced by the software: the x-axis is time-dependent, with green segments indicating the moments of proper pressure and red segments the incorrect ones. The y-axis shows the EI values for the DJC (yellow) and DDET (green). The values of EI are expressed at the top right side of the image. SR, not shown, is then calculated by the software (ROI: region of interest; DJC: distal insertion of the joint capsule; DDET: dorsal digital extensor tendon; EI: Elasticity Index; SR: Strain Ratio)

Transverse and longitudinal elastosonographic scans showing the positioning of the ROIs over the DJC and the DDET for the qualitative and semi-quantitative analysis in one horse of Group S and in one horse of Group P are displayed in Figs. 3 and 4 a, b.

In both groups, significant differences were not observed between or within operators. In Group S, the inter-CC for EI in the transverse and longitudinal scans was 0.94, and that for SR was 0.84–0.92. The intra-CC for EI in transverse and longitudinal images was 0.98 − 0.97, and that for SR was 0.98 − 0.95.

In Group P, the inter-CCs for EI in transverse and longitudinal scans were 0.57-0.94, and for SR, they were 0.69–0.92. The intra-CC for EI in transverse and longitudinal images was 0.94-0.94, for SR 0.81–0.96.

The inter-CC and intra-CC of EI and SR in both scans for Group S and Group P are summarized in Table 3.

**Table 3:** Inter-CC and intra-CC of EI and SR in transverse and longitudinal scans for Group S and Group P (*inter-CC* interclass correlation coefficient, *intra-CC* intraclass correlation coefficient, *EI* elasticity index, *SR* strain ratio)

		Transverse Scan	Longitudinal Scan
**Group S**	**Inter-CC of EI**	0.94	0.94
	**Intra-CC of EI**	0.98	0.97
	**Inter-CC of SR**	0.84	0.92
	**Intra-CC of SR**	0.98	0.95
**Group P**	**Inter-CC of EI**	0.57	0.94
	**Intra-CC of EI**	0.94	0.94
	**Inter-CC of SR**	0.69	0.92
	**Intra-CC of SR**	0.81	0.96

Significative differences in EI and SR values were detected between Group S and Group P in both scans. When Group S was compared to the affected limb of Group P, EI and SR were significantly lower for Group S. When Group S was compared to the unaffected limb of Group P, EI and SR values were significantly lower in Group S only in longitudinal scans.

No differences were detected for EI and SR values between the affected and unaffected limbs in Group P.

No significant differences between the right and left limbs were observed in either group. Significant differences were not observed between female and male horses.

## Discussion

Chronic inflammation of the articular capsule is recognized as one of the primary mechanisms for cartilage damage, changes in tissue elasticity, and fibrosis of the joint capsule and soft tissue surrounding the joint [4]. Since elastosonography determines tissue elasticity by measuring the degree of strain that tissues experience in response to external stress [3], we aimed to show whether SE was a feasible technique for evaluating DJCs in sound horses (Group S) and in horses with osteoarthritis (Group P).

We first endeavoured to assess whether the technique was feasible and able to produce repeatable results. For the qualitative and quantitative analysis, no significant difference was found between measures taken on the same structure by two different operators or between repeated measures from the same operator (*P* > 0.05). However, an assessment of IRR and ICC revealed that sound horses had better reproducibility and repeatability compared to those with OA (Group P), with higher values for both parameters; horses with OA had a higher variability of the elastosonographic pattern of the DJC, probably due to different stages of the pathologic process in these joints.

Concerning the qualitative analysis, we performed a colour grading score assessment of DJC with five degrees of judgement, although previous studies utilized a different colour grading score, with seven degrees of judgement; we decided to use a simpler scale, which could be more applicable for clinical use [6]. The qualitative appearance of the DJC was slightly softer in the longitudinal scans than in the transverse scans (4.95 vs. 4.79 in Group S and 3.78 vs. 3.68 in Group P). The longitudinal scans also showed more reliability, potentially due to the anisotropy of musculoskeletal tissues or the increased number of artefacts on the lateral and medial sides of the transverse ultrasonographic image [16–18]. To reduce this variability attributed to inhomogeneous application of pressure, the lateral and medial aspects of the US images were avoided when the ROI was selected. A significant difference was detected between Group S and Group P when these were considered as a whole sample (*P* < 0.05) and when Group S was compared to the affected limbs and to the unaffected limbs of Group P. We suppose that the difference between Group S and the unaffected limb of Group P can be ascribed to subclinical degenerative changes in the contralateral “sound” joint of this group. However, the lack of further information (MRI, arthroscopy, histology) makes it difficult to confirm this statement. Most importantly, a significant difference was detected between the affected and unaffected limbs of Group P (*P* < 0.05), suggesting different elastic features between the affected and unaffected capsules of the same horse.

Discussing the results of the quantitative analysis, as previously described in studies of tendon elastography in humans and in horses, it was determined that the EIs and SRs were lower for longitudinal scans than for transverse scans [6–9, 17]. EI and SR values were significantly lower in Group S than in Group P in both longitudinal and transverse scans.

The DDET was chosen as the reference tissue because it was expected to experience the same degree of strain as the DJC. The ROI was consistently placed at the level of the metacarpophalangeal articular space to facilitate a similar comparison of the target region with the same area of the DDET. However, the lack of elastographic data about the DDET could be responsible for the high standard deviation that was observed.

The choice of the ROI is critical for successful SE, especially when large structures are examined or when no clear lesions are visible on B-mode ultrasonography. In this study, only a small ROI was required. In cases where larger structures are examined, care should be taken when analysing results, and a computerized analysis of the percentage of pixel distribution may be needed [6].

Even though mild sedation for research purposes was provided to all patients, this may not be necessary in clinical practice when horses are calm and standing still.

Previous studies have reported the use of a standoff pad to increase the distance between the skin and the probe, as a specific minimum distance from the skin (usually 1.2 mm) is needed to place the elastogram box [6, 14]. Although both DJC and DDET are superficial structures, the distance from the probe was enough to place the box of the elastogram in all of the horses examined in our study without using any standoff device. We decided not to use a standoff pad because we found that it was more difficult to apply the correct amount of pressure on the structure with the pad and, simultaneously, to avoid axial motion of the US probe with incomplete secondary contact with the standoff device. Moreover, the use of pads may generate artefact occurrence (i.e., reverberation artefacts), which could be mistaken for regions of softness [6].

We successfully evaluated the distal insertion of the metacarpophalangeal joint capsule in horses of different breeds and ages. Although we selected horses according to the radiographic and ultrasonographic signs of OA, the wide age distribution of the admitted patients can be considered another limit of the study, as different elastic features of tissue can be supposed between young and older horses. Moreover, the lack of magnetic resonance or histology of the examined structures did not allow us to confirm our hypothesis about the different tissue compositions, so we can only suppose that the difference between the samples is an effect of the variations.

Based on the radiographic and ultrasonographic scores, the sample of horses of group P was affected by a mild to moderated degree of OA. Since we selected horses from a population of spontaneously incoming patients to the Veterinary Teaching Hospital, this data could have been biased. Riding horses were the most represented type of referred horses, which are not usually affected by a severe degree of degeneration of the joints, differently from racing horses, which often display more severe joint conditions at older ages [19].

From the data analysis, it can be said that the technique was feasible in both groups of horses, sound and suffering from fetlock OA, although higher variability of data was found in patients with radiographic and ultrasonographic signs of OA. We also demonstrated that sound horses had an elastogram suggestive of softer DJC compared to the other group.

Although the procedures were not difficult to perform, reproducibility of the techniques for scanning tissues, reading elastograms, and storing data should be evaluated in less skilled operators. Further studies should investigate the effects of age and breed on the appearance of this anatomical landmark.

## Conclusions

This study showed that strain elastosonography is a useful technique for evaluating the distal insertion of the metacarpophalangeal joint capsule in horses. We think that this approach can be used as a complementary diagnostic technique to B-mode ultrasonography to monitor the response to treatment or the evolution of rehabilitation programmes in joints suffering from OA. Strain elastosonography showed good repeatability and reproducibility and high reliability, especially in qualitative assessments. Future studies should focus on the establishment of a relationship between different degrees of osteoarthritis and elastographic features of DJCs in horses.

## Supplementary Information

**Additional file 1. **Supplementary material: the table shows theultrasonographic score, modified from Yamada etal, 2020.

## Data Availability

The datasets used and/or analysed during the current study are available from the corresponding author on reasonable request.

## Abbreviations

DDET: Dorsal digital extensor tendon
DJC: Distal attachment of the joint capsule
EI: elasticity index
Inter-CC: Interclass correlation coefficient
Intra-CC: Intraclass correlation coefficient
IRR: Inter-rater reliability
OA: Osteoarthritis
P1: Proximal phalanx
ROI: Region of interest
SE: Strain Elastography
SR: Strain Ratio
