# Supplementary material for: Elastosonographic features of the metacarpophalangeal joint capsule in horses
Source: BMC Vet Res. 2021 May 29;17:202. doi: 10.1186/s12917-021-02897-8 (PMC8164309; doi:10.1186/s12917-021-02897-8)
Supplement: Supplementary file 1 — Additional file 1. Supplementary material: the table shows theultrasonographic score, modified from Yamada etal, 2020. [file 12917_2021_2897_MOESM1_ESM.docx]

| Variables | | Categories | | Score |
| --- | --- | --- | --- | --- |
| Subchondral surface appearance | | Smooth | | 0 |
|  |  | Irregular | | 1 |
|  |  | Areas of depression | | 2 |
| Synovial plica | Appearance | | Normal | 0 |
|  |  |  | Predominantly hyperechogenic | 1 |
|  |  |  | Hyperechogenic calcification sites | 2 |
|  | Size | | Normal | 0 |
|  |  |  | Increased up to 50% | 1 |
|  |  |  | Increased by more than 50% | 2 |
| Joint capsule | Insertion | | Smooth | 0 |
|  |  |  | Discretely irregular | 1 |
|  |  |  | Irregular | 2 |
|  |  |  | Severe irregularity | 3 |
|  | Appearance | | Normal | 0 |
|  |  |  | Localized hypoechogenic foci | 1 |
|  |  |  | Hypoechogenic areas with hyperechogenic foci | 2 |
|  | Thickness | | Normal | 0 |
|  |  |  | Increased by 20% in localized areas | 1 |
|  |  |  | Increased | 2 |
|  |  |  | Increased by more than 20% | 3 |
| Total (final sum) | | | | 14 |

Supplementary material: the table shows the ultrasonographic score, modified from Yamada et al, 2020.
